# Effect of Margin Design and Processing Steps on Marginal Adaptation of Captek Restorations

**DOI:** 10.5402/2011/810565

**Published:** 2011-06-08

**Authors:** Amy Shih, Robert Flinton, Jayalakshmi Vaidyanathan, Tritala Vaidyanathan

**Affiliations:** Department of Restorative Dentistry, New Jersey Dental School, UMDNJ 110 Bergen Street, Newark, NJ 07103, USA

## Abstract

This study examined the effect of four margin designs on marginal adaptation of Captek crowns during selected processing steps. Twenty-four Captek crowns were fabricated, six each of four margin designs: shoulder (Group A), chamfer (Group B), chamfer with bevel (Group C), and shoulder with bevel (Group D). Marginal discrepancies between crowns and matching dies were measured at selected points for each sample at the coping stage (Stage 1), following porcelain application (Stage 2) and cementation (Stage 3). Digital imaging methods were used to measure marginal gap. The results indicate decreasing trend of margin gap as a function of margin design in the order A>B>C>D. Between processing steps, the trend was in the order Stage 3 < Stage 1 < Stage 2. Porcelain firing had no significant effect on marginal adaptation, but cementation decreased the marginal gap. Generally, the margin gap in Captek restorations were in all cases less than the reported acceptable range of margin gaps for ceramometal restorations. These results are clinically favorable outcomes and may be associated with the ductility and burnishability of matrix phase in Captek metal coping margins.

## 1. Introduction

Materials used for crown and bridge applications include metals, ceramics, and metalceramics. Although metals have excellent mechanical properties, full metal crowns are not generally preferred due to poor esthetics. All-ceramic restorations are now used for single crowns and possibly short span bridges because of their improved strength and esthetics. While ceramics are generally abrasive to opposing dentition, the high strength all-ceramic restorations may cause even more wear [1]. Ceramometal restorations, which combine esthetics and fracture toughness, are, therefore, often preferred for crown and bridge applications in general and in clinical situations where high toughness is desired (e.g., long-span bridges or in patients with bruxism and other parafunctional habits), they may potentially be the materials of choice. Ceramometal restorations are fabricated as porcelain fused-to-metal (PFM) copings. However, even in high gold ceramic alloys, addition of elements such as platinum (Pt) and palladium (Pd) used to strengthen or optimize alloy coefficient of thermal expansion results in loss of alloy gold color. When such restorations with bare metal margins are placed subgingivally, the gray color may show through the gingival margins, especially in patients with tissue of the thin biotype. A more esthetic ceramogold restoration system was developed in the last decade to overcome the problem of gray show-through [2]. This system called Captek (named after capillary technology used in its processing steps) uses what is described as a composite metal structure in which a gold-rich matrix alloy is embedded with a particulate metallic phase during processing to provide both esthetics and strength. Captek alloy (Precious Chemicals Co., Inc., Altamonte Springs, Fla, USA) has two main components: Captek “P” and Captek “G” [2]. They come in elastic wax sheets impregnated with metallic particles and having an average thickness of about 0.3 mm for crown application. They are both manually modeled to form crown copings over refractory dies. The Captek P is processed first and fired in a porcelain furnace. The firing temperature is lower than melting temperature of the alloy particles. Consequently, instead of melting, the particles undergo sintering (a process of welding together of particles at a temperature below the melting point of the powder). This procedure is used to form a porous skeleton of the coping. Captek G is then applied over the Captek P and fired again, to complete the coping. The alloy particles used in Captek G melts completely, flows into the capillary pores of the fired Captek P scaffold, and forms a matrix embedding the alloy particles of Captek P after the firing cycle. The average composition of the composite alloy is listed by the manufacturer as about 88% gold (Au), 4.2% platinum (Pt), and 4.5% palladium (Pd) [2]. Based on an analysis of the Captek P and G component alloy fractions by Zappala et al. [3], the P component contains microflakes of 39.4% Au-29% Pt-30.2% Pd-2.4% Ag alloy, while the G-component alloy contains atomized gold with 2% Ag. An esthetic matrix rich in Au is thus formed with the microflake particles (partially dissolved into the matrix ensuring structural continuity) serving as reinforcing dispersed phase to strengthen the alloy matrix. When this system is used to make PFM restorations, the metal margins placed subgingivally in thin biotype tissue appears in esthetic gold color of the matrix, satisfying patient demand for improved esthetics. 

In the past, traditional ceramometal restorations have been studied extensively to assess their in vitro and in vivo performance [4–6]. Although Captek PFM restorations have also been extremely popular among many practitioners, there are only a limited number of published studies, and these studies were focused on different topics such as processing details [2], metallurgical properties [2, 3], bonding to porcelain [7], esthetics [8], antiplaque properties [9], and mechanical properties [10, 11]. Marginal fit properties were also reported [10, 12], but the controlled variables were limited to demonstrate that the accuracy of the finished crown fit was within acceptable range. Thus, although the improved esthetics of Captek over traditional PFM restorations is now well recognized, there is, nevertheless, a dearth of systematic studies on marginal adaptation of Captek restoration as it undergoes changes during different processing steps. Successful marginal adaptation of PFM systems is not only dependent on the ability of an operator in preparing tooth and the skill of the technician in fabricating restoration, but also on the nature of the metal used to make the restoration and its response during the porcelain veneering and cementation steps. Different margin designs used clinically may also impact upon margin adaptation. Since poor marginal adaptation may lead to recurring caries [13] as well as possible increase in periodontal inflammation [14], a systematic study of the marginal fit of Captek system under different margin design conditions and processing steps is the main objective in this study. The hypotheses in this study were designed to test whether the crown restorations fabricated from Captek system satisfy the range of clinical acceptability of fit at the margins and to assess the role of margin design as well as porcelain firing and cementation steps on margin gap formation.

## 2. Materials and Methods

Four ivorine typodont teeth (upper left central incisor) were prepared by one operator and mounted into a typodont. One each was prepared to exhibit following margin designs: shoulder (A), chamfer (B), chamfer with bevel (C), and shoulder with bevel  (D). All preparations were made using Brasseler medium-grit diamond burs followed by fine diamond burs to provide smooth finished margins. The preparations followed conventional guidelines for PFM restorations leaving space for 0.5 mm thickness of metal and 1 mm thickness of porcelain. 

 Marginal gap measurements were made along the coping margins fitted on dies (fabricated from the typodont tooth) after each stage of processing, namely, (a) after Captek metal coping fabrication (b) after porcelain firing, and (c) after crown cementation.

A repeated measures ANOVA design was used with four subjects (margin designs) and three within subject variables (metal, porcelain fused to metal, and cemented copings). Power analysis (*α* = 0.05, *β* = 0.10) was used to select a sample size of *N* = 6/group.

### 2.1. Die Preparation from Typodont Tooth

**Figure 1:**
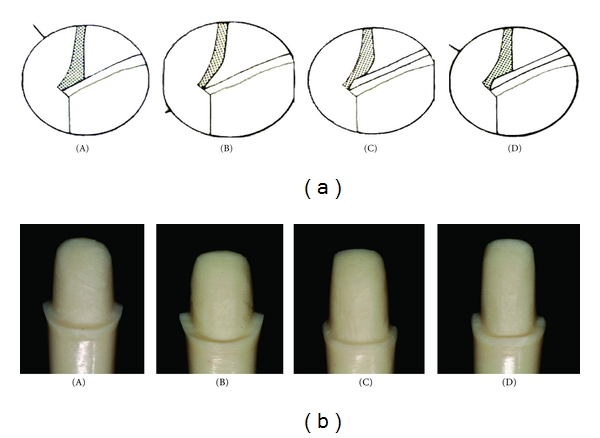
(a) Exploded line illustration of margin designs of (A) shoulder (B) Chamfer (C) Chamfer with bevel) and (D) shoulder with bevel. (b) Actual resin dies fabricated from typodont tooth prepared to follow corresponding margin designs illustrated in Figure 1(a).

An Ortho Resin (Dentsply/Caulk, Milford, Del, USA) base was fabricated into which the typodont tooth was placed so that each die would exhibit a base of similar size and shape. Four groups of six impressions of each prepared tooth were made using polyvinyl siloxane (PVS) impression material (Examix, GC America, Alsip, Ill, USA) in plastic dispensing cups. Prepared tooth was injected with light body PVS around the margins and inserted into medium body PVS placed in the cup. The tooth was carefully removed from the impression after setting for four minutes. All impressions were carefully checked to be free of bubbles or defects. Epoxy resin (Die Epoxy, American Dental Supply, Inc., Allentown, Pa, USA) was poured into each impression and was spun in a centrifuge to minimize air bubbles. After setting for 24 hours, the die was carefully separated from impression and checked to be free from bubbles and defects. New resin dies were prepared to replace any defective ones.  Figure 1(a) shows typical dies made with different margin designs illustrated in Figure 1(b). 

A set of four groups of six resin dies were thus obtained, and these dies were used for actual marginal gap measurements during different processing stages (i.e., before porcelain firing on metal coping, after porcelain firing, and after cementation).

Using the original impressions, four groups of six stone dies from Type IV stone (Die-Keen, Modern Materials, Heraues Dental North America, South Bend, Ind, USA) were also prepared. The stone dies were retrieved from the impressions by cutting away the impressions and checked to be free of bubbles and defects, and any die with defects was remade using a new impression. When any stone die was discarded, the original resin die was also discarded, and a new resin die was fabricated to replace the discarded die.

### 2.2. Coping Fabrication Steps and Gap Measurement Sequence

The set of stone dies prepared were used to make new refractory dies for making Captek copings. All copings were prepared by a dental technician certified in Captek System. Manufacturer recommended procedures were followed in the coping fabrication and subsequent processing steps. First, Captek adhesive was applied to the refractory dies and allowed to dry, followed by manual adaptation of the coping shape on the refractory die using Captek P strip. Excess material at the margin was removed and the refractory die with the Captek P coping layer was processed as per the recommended firing cycle (preheat temperature 600°C, heat to 1075°C at 80°C/min, and hold 4 minutes in vacuum). This initial processing step creates a sintered porous scaffold in the shape of the coping. An overlay of the Captek G elastic strip was again contoured and placed on top of the P-layer and refired using the same firing cycle to complete the coping fabrication. The copings were seated on the resin die prepared initially, and the gap between the coping margin and the resin die was measured in a Bioquant digital imaging system, as detailed in a protocol described later. After gap measurement, the coping was subjected to porcelain firing to make PFM restorations. An index was fabricated for porcelain application similar to the one used by Shillingburg et al. [15]. The index was made by adapting resin around original tooth. This was used to apply porcelain to the copings and achieve crowns of the same height, width, and buccal and lingual contours. The firing cycle included three porcelain firing steps (opaque, body, and glaze). After firing, the marginal gap was again measured according to the protocol to be described. 

Following gap measurements, the copings were then cemented to resin dies under standardized conditions (250 N compressive load applied in a mechanical Testing machine for four minutes on the incisal edge of the PFM crown mounted on a jig to hold it upright. 

Reinforced glass ionomer (GC Fuji Plus, GC America) cement was used for cementation. The standardized load was applied in a MTS machine model 810 (Mechanical Testing Systems, Inc. Eden Prairie, Minn, USA) working under programmed load control. This was done to remove load as a variable affecting marginal gap. Excess cement was removed with an explorer once the cement reached the initial gel stage as per the manufacturers' instructions. The marginal gaps were again measured using the protocol described below.

### 2.3. Marginal Gap Measurement Protocol

**Figure 2:**
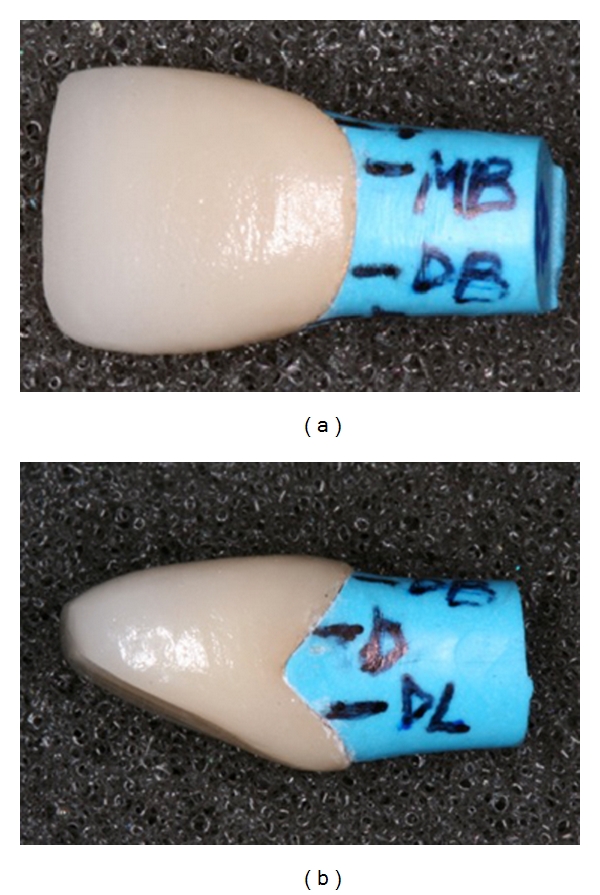
Reference points marked on the die for repeatable margin gap measurements at the same locations after different processing stages. (a) Buccal view. (b) Distal view.

Each coping was examined on its corresponding resin die to verify visually by inspection that the fit was acceptable. The marginal adaptation was measured at selected points around the circumference of each sample under a stereo light microscope (SteroeZoom 4, Bausch and Lomb, Rochester, NY, USA). Five points were identified on each resin die; one point at which the coping-die interface appeared “closed” under magnification, and four points at which distinct gap openings were observed and measured. All points for measurement were selected in the first stage during gap measurements with the Captek metal copings before porcelain firing. A fine tip permanent marker was used to mark a line corresponding to the points of interest but not extending onto the margin of the die to keep the margin area clearly visible (Figure 2).

**Figure 3:**
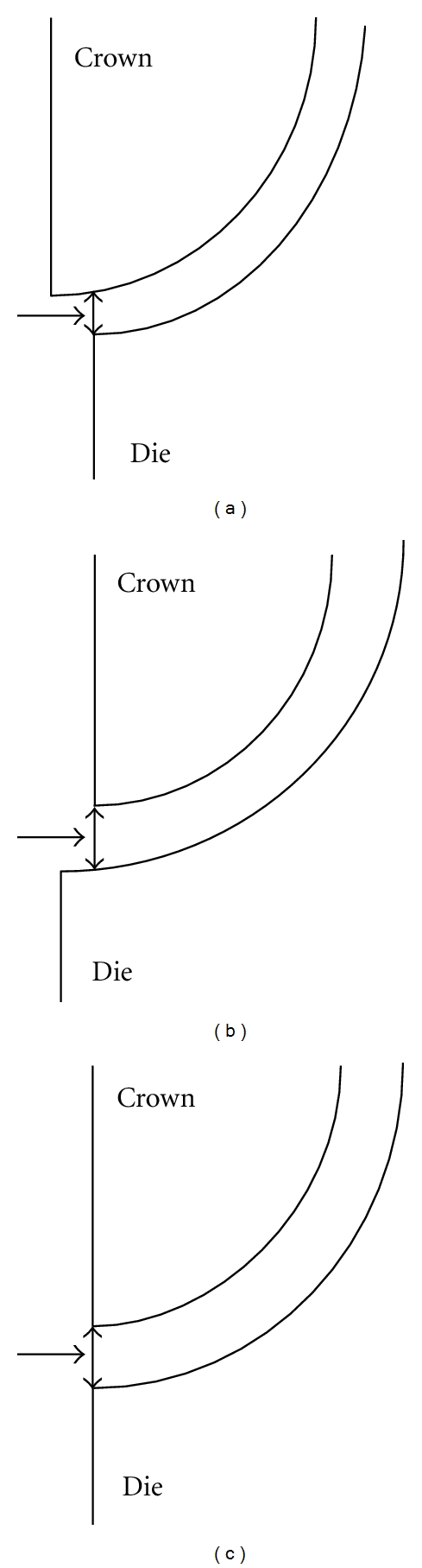
Measurement of vertical marginal discrepancy for cases involving (a) overextended (b) underextended, and (c) no over- or under- extended conditions.

This facilitated measurement at the same points for subsequent stages (after porcelain firing and after cementation). The gaps were measured using a Bioquant Imaging System (Bioquant Nova, Bioquant Image Analysis Corp., Nashville, Tenn, USA) interfaced to the microscope set at 45x magnification. The system uses digital imaging techniques to capture and quantify dimensional details using gray level contrast of pixels in the image. The number of pixels along gap width and the pixel size are used to estimate margin gap. The microscope was calibrated with a 1 mm × 1 mm calibration slide (under the preset magnification) prior to any images being captured. The magnification established during calibration was maintained constant by adjusting coping-fitted die marginal gap area position to the preset focal plane of microscope during measurement. In addition, any parallax error was minimized by using a reproducible seating arrangement of the coping-fitted die on the microscope stage. Following capture of the images at selected points, gaps were measured as shortest straight line distances from a selected point on the die to the coping edge through the margin gap, as illustrated in Figure 3. When viewed in the direction (see arrows in Figure 3) shown, this corresponds to the vertical marginal discrepancy described by Holmes et al. [16] for different situations of (a) overextended coping, (b) underextended coping, and (c) where finish line of die is aligned to edge of the coping margin. Measurements were done for each of four premarked points, after allowing for magnification.

### 2.4. Statistical Analysis

Data was analyzed using repeated measures ANOVA and post hoc Tukey contrast to identify significant differences between subjects (margin designs) and between within subject variables (stages). One-way ANOVA and Tukey contrasts were used to differentiate differences between margin designs within each stage. All Significant differences were assessed at *P* <  .05. SPSS program version 15 was used in the analysis.

## 3. Results

**Figure 4:**
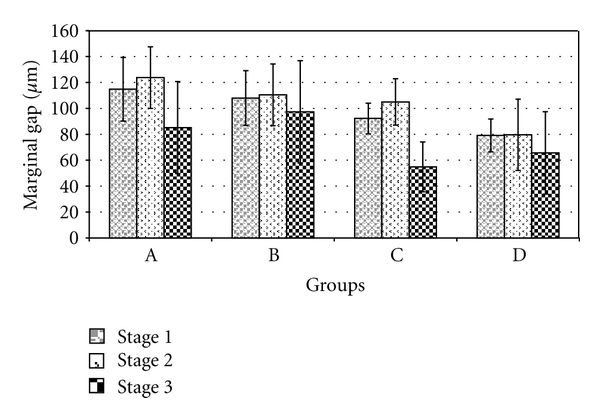
Bar graph of marginal gap variation as a function of margin design differences and processing stages.

Figure 4 is a bar graph of the means and (SD) of the marginal gap data as a function of marginal design and processing stages. The measured mean and (SD) of margin gap (*μ*m) ranged from 54.85 (19.16) for Group C, (stage 3) to 123.70 (23.74) for Group A (stage 2). Differences in margin gap means were observed both as a function of margin design (between groups) and stages (within groups). 

Repeated measures ANOVA results are presented in Table 1. Significant differences in gap means are observed between margin design differences (*P* <  .001). Margin gaps increased in the order A > B > C > D for different groups. The results suggest that chamfer and shoulder designs reduce margin gap in the presence of bevels. Significant differences are also noted between processing stages (*P* = .002). Margin gaps decreased in the order stage 3 < stage 1< stage 2. Porcelain firing (stage 2) on the metal coping appears to cause an increase in margin gap, while cementation (stage 3) reduces the gap. There was no significant group∗stage interaction effect (*P* = .630).

One-way ANOVA and Tukey comparisons as a function of margin design groups within each processing stage are presented in Table 2. Significant differences between groups are identified in stages 1 (*P* = .014) and 2 (*P* = .018). Within stage 3, the results support null hypothesis showing no significant differences between groups (*P* = .135). During cementation (stage 3), the margin gaps are lower in all groups. 

**Figure 5:**
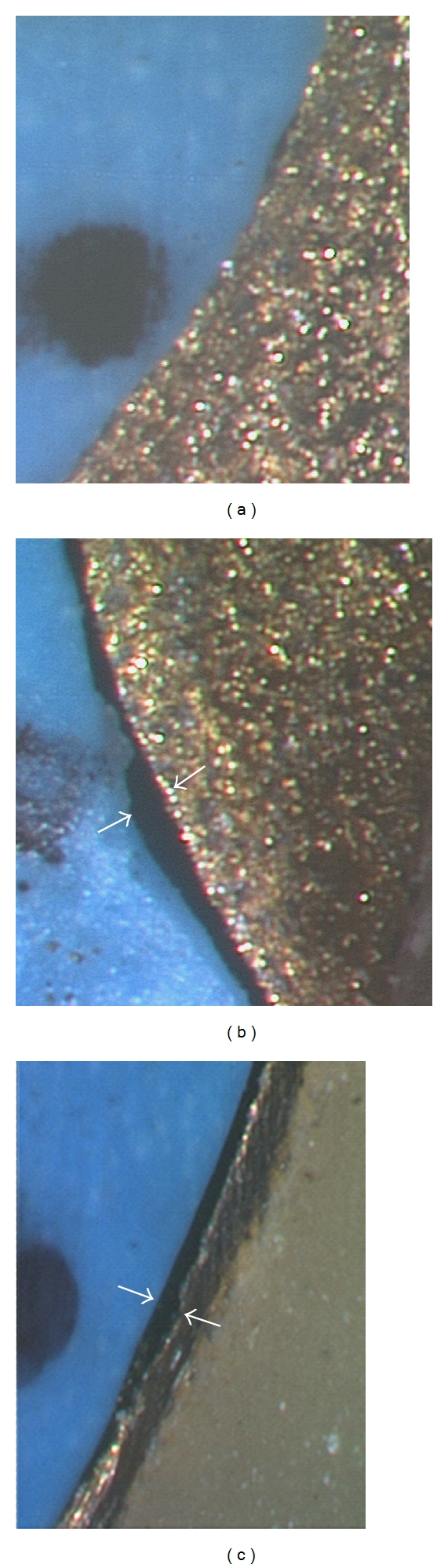
Marginal gap displayed in captured images. (a) Example of “closed” marginal gap. (b) Example of “open” marginal gap. (c) Example of marginal gap next to metal collar. Gaps width is identified by arrows in the respective figures.

Figures 5(a), 5(b), and 5(c) show examples of captured images representing closed and open margins and the open margins visible as dark bands near the die-coping margin. In Figure 5(b), the margin shows no gap, but in Figures 5(a) and 5(c), clear gaps at the die-coping margins are observed. In Figure 5(c), the gaps are seen right next to the metal collar. Thus, the fit of the coping on the die demonstrated differences on different samples.

## 4. Discussion of Results

Poor marginal fit is recognized to be a potential source of long-term failure of metal ceramic restorations necessitating their replacement. The initial fit of restorations is, therefore, a critically important consideration in assessing their acceptability. The fit is typically measured by the gap at the margins. Although margin gaps can be measured under different conditions, the method of vertical marginal discrepancy defined by Holmes et al. [16] was used in this study. 

There is no firm consensus on what constitutes a clinically acceptable crown fit. However, based on analysis of criteria used by experience practitioners, margin gaps as high as 120 *μ*m are considered clinically acceptable (Christensen [17], McLean and Von Fraunhoffer [18], and Dedmon [19]). Based on this criterion, Captek system typically demonstrated clinically acceptable range of marginal adaptation in general and well below maximum acceptable value after cementation. 

In this study, the first objective was to evaluate differences in the marginal fit of Captek restorations when the restoration is processed under different marginal designs. Our results indicate that restorations with bevels showed lower margin gaps in stages 1 and 2 in all cases, but there was no statistically significant difference after cementation. Faucher and Nicholls [20], Shillingburg et al. [15], and Preston [21] have previously reported that shoulder margin design performed better than the chamfer in traditional PFMs. This is not the case in our study of Captek system. It is possible that the heterogeneity in a two-phase composite structure with a softer matrix may alter the margin behavior, because margins may be predominantly made up of the softer matrix alloy in Captek system. 

It was also found that the margin gap means generally increased with porcelain firing on the coping, but the difference was not statistically significant (see Table 1). Previous reports in the literature have shown either an increase [4, 5] in marginal gap or no significant effect during porcelain firing [6]. Finite element modeling by DeHoff and Anusavice [22] has indicated that the stresses generated during porcelain firing are unlikely to cause predictable distortion at the margin in PFM alloys. However, these calculations were done in alloys with high modulus properties. The results in this study confirm that even in Captek system, where the margin composition may be primarily that of softer gold-rich metallic matrix form component G, porcelain firing causes no significant change in margin distortion.

**Figure 6:**
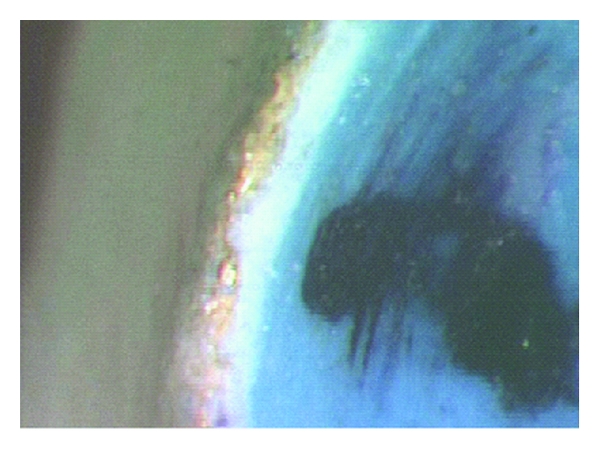
Illustration of typical margin after cementation. Note the effect of marginal burnishing during excess cement removal.

Results of one-way ANOVA indicate that for stages 1 and 2, there was a significant difference between different margin groups, but for stage 3, the difference between groups is nonsignificant. This is in keeping with the findings of Hamaguchi et al. [23] for traditional PFM alloys. Interestingly, while there was no significant difference between margin groups in stage 3, the mean margin gap values in this stage were also significantly lower than in other stages. This finding is of major importance in that this stage is of most relevance in a clinical situation. It is to be noted that in traditional PFMs, this is not the case. During stage 3, the excess cement is mechanically removed. During this mechanical removal, the soft metal margins in Captek may potentially be burnished and closed up for a better fit. This is illustrated by the image of a cemented margin in Figure 6. Even though the margins are not intentionally burnished, cement removal may work as a quasiburnishing step. In traditional PFM alloys, margins are stiffer and are unlikely to be burnished during excess cement removal. 

Thus, the results of this study suggest that the softer alloy matrix phase of Captek system may influence the fit at the margins even though significant similarities exist in the manifestation of margin gaps in Captek system and traditional PFM systems.

## 5. Conclusions

The following major conclusions were drawn from this study.

(i) Marginal discrepancy in Captek restorations fall within acceptable clinical criteria used by prosthodontics practitioners.

(ii) Margin design with bevels exhibited less marginal discrepancy than those without bevels.

(iii) The marginal discrepancy was typically higher for crowns before and after porcelain firing but was significantly less after cementation. During mechanical removal of excess cement, the crown margins may undergo burnishing and close up the gap.

## Figures and Tables

**Table 1:** Repeated measures ANOVA results and post hoc Tukey comparisons.

	*n*	Mean	SD	*P *value	Tukey post hoc*	
Stage 1	24	94.48	3.75	.002	A	
Stage 2	24	104.66	4.79		A	
Stage 3	24	75.73	6.61			B

Group A	6	107.67	5.08	<.001	A	
Group B	6	105.20	5.08		A	B
B Group C	6	83.97	5.08		A	B
Group D	6	74.78	5.08			B

Stage∗Group	—			.630	N/A	

*letters A and B indicate homogenous subsets, respectively.

**Table 2:** Summary of one-way ANOVA with Tukey post hoc comparisons.

	Group	*n*	Mean	SD	*P *value	Tukey post hoc*	
Stage 1	A	6	114.77	24.57	.014	A	
	B	6	107.93	21.10		A	B
	C	6	92.13	11.89		A	B
	D	6	79.11	12.67			B

Stage 2	A	6	123.70	23.74	.028	A	
	B	6	110.39	23.84		A	B
	C	6	104.94	17.65		A	B
	D	6	79.61	27.48			B

Stage 3	A	6	85.15	35.45	.135	N/A
	B	6	97.28	39.46		
	C	6	54.85	19.16		
	D	6	65.63	31.77		

*letters A and B indicate homogenous subsets, respectively.
